# Predictors of stunting among children 6–59 months of age in Sodo Zuria District, South Ethiopia: a community based cross-sectional study

**DOI:** 10.1186/s40795-019-0287-6

**Published:** 2019-03-11

**Authors:** Samson Kastro Dake, Fithamlak Bisetegen Solomon, Tesfahun Molla Bobe, Habtamu Azene Tekle, Efrata Girma Tufa

**Affiliations:** 1 College of Health Sciences and Medicine, School of Public Health, Department of Reproductive Health and Nutrition, Wolaita Sodo University, Sodo, Ethiopia; 2 College of Health Sciences and Medicine, School of Medicine, Department of Medical Laboratory, Wolaita Sodo University, Sodo, Ethiopia; 3 College of Health Sciences and Medicine, School of Medicine, Wolaita Sodo University, Sodo, Ethiopia

**Keywords:** Stunting, 6–59 months, Child, Predictors, Sodo-zuria

## Abstract

**Background:**

Despite the decline in the rate of stunting in Ethiopia, the prevalence is still high and needs immense efforts to achieve the target set to reduce the prevalence. It varies between localities due to individual level factors and dominant livelihood practice in the community.

Thus, the aim of this study was to determine the prevalence of stunting and identify factors associated with it in Sodo Zuria district in South Ethiopia.

**Methods:**

A community based cross sectional study was conducted among 342 children aged 6–59 months paired with mothers/caretakers. Households were selected using systematic sampling. Structured questionnaire was used and mothers/caregivers were interviewed face to face. Standardized anthropometric measurements were used to measure length, and weight and height of a child. Data were entered into Epi Info software version 3.5.1 and exported to SPSS version 20 for analysis. Height for age Z score data were analyzed using WHO Anthro software. Multivariate logistic regression analysis was conducted to identify predictor variables. Statistical significance was considered at *p* < 0.05.

**Results:**

The prevalence of stunting in this study was 24.9% with 7.9% being severely stunted. Being female (AOR = 2.8; 95% CI: 1.5, 5.3), children aged 12–23 months (AOR = 7.1; 95% CI: 2.3, 21.9), mother’s who do not use family planning (AOR = 2.5; 95% CI: 1.1,5.7), children with diarrheal morbidity (AOR = 2.5; 95% CI: 1.2,5.3), income of 750–1500 ETB and > 1500, and children who received pre-lacteal feeding (AOR = 3.8; 95% CI: 1.2–12.2) became predictors for stunting.

**Conclusion:**

Significant proportion of stunting was found where one third of them were severely stunted. Being female, children aged 12–23 months, using family planning, children with diarrheal morbidity, income and pre-lacteal feeding became predictors for stunting. So Gender-based policies should be enacted in child feeding practice, interventions should focus on the utilization of family planning and appropriate child caring and feeding practices. Water, sanitation and hygiene interventions need to be strengthened.

## Background

Stunting (low height-for-age) is a sign of chronic undernutrition that reflects failure to receive adequate nutrition over a long period. Children are regarded as stunted when their height-for-age Z-score (HAZ) is below minus two standard deviations (−2SD) from the median of the reference population [1]. Stunting reflects failure to get adequate nutrition over a long period of time and it can be affected by recurrent and chronic illness [2].

Malnutrition leads to serious public health and economic risks, and improvements in nutrition will contribute significantly to reducing poverty, and to achieving health, education, and employment goals [3]. Child undernutrition is highly prevalent in low and middle-income countries and substantially increases mortality and overall disease burden [4]. Globally, 161 million (25%) children under-5 years of age were estimated to be stunted and one third of all stunted children lived in Africa [2]. The highest prevalence of stunting is reported in South Asia (36%) and Sub-Saharan Africa (34%) [5]. In Ethiopia the prevalence of stunting is 38% [1], ranking the country among the top both in sub Saharan Africa and the World.

The World Health Organization [WHO] adopted a resolution with a target to reduce the number of stunted children under-five by 40% in 2025. However, at current rates of progress, stunting level will decrease by 26% in 2025 [6]. Similarly, despite the decline in the rate of stunting from 47% (2005) [7] to 38% (2016) [1] in Ethiopia, the prevalence is still high and needs immense efforts to achieve the target set to reduce the prevalence to 26% in 2020 [8]. Childhood stunting varies between localities due to individual and community-level factors [9, 10] and the factors associated with stunting vary by the dominant livelihood practice in the community [11]. However, there are no documented studies to identify the factors associated with stunting in the study area. Thus, the aim of this study was to determine the prevalence of stunting and identify factors associated with it in Sodo Zuria district in South Ethiopia.

## Methods

### Study design and setting

A community based cross sectional study was conducted from May to June 2017. Sodo Zuria is 380 km south of Addis Ababa and it has 30 kebeles (lowest administrative units) with a projected population of 394,772; 24,647 were children 6–59 months of age.

### Population and sampling

The study population were randomly selected children, 6–59 months of age paired with their mothers/caregivers who resided in the district for at least 6 months. Children with physical disability who are not eligible for anthropometric measurements were excluded from the study. A sample size of 380 was calculated with 95% confidence level, 5% margin of error, an expected stunting prevalence of 38% [1] and non response rate of 5%. Sampling frame was developed for the randomly selected 4 kebeles and the total sample was proportionally allocated to the selected kebeles. Systematic sampling was used to select households and a child was selected randomly in households with more than one eligible children.

### Variables

#### Outcome variable

Stunting which was computed with height for age z-score (HAZ) of less than − 2 standard deviation (SD).

#### Exposure variables

Socio demographic and economic variables: Maternal age, marital status, religion, ethnicity, maternal education, paternal education, maternal occupation, paternal occupation, household income in Ethiopian birr (ETB), family size, decision making on household resource utilization and age of the child.

Obstetric and other maternal characteristics: Place of delivery, gestational age at birth, birth weight, birth order, pregnancy interval, age at first birth, antenatal care (ANC) follow up, one or more additional meal consumption than usual and utilization of family planning.

#### Child morbidity

Diarrhea, fever, acute respiratory infections and measles.

#### Child caring practice

Breast feeding, pre lacteal feeding, complementary feeding, dietary diversity and child vaccination.

#### Environmental health characteristic

Source and distance of drinking water, availability of latrine, hand washing practice, household waste disposal.

### Data collection

Data collectors with prior experience of data collection interviewed mothers/caregivers face to face using pretested and structured questionnaire. The data collectors were trained on the procedures of data collection and were standardized on obtaining anthropometric measurements. A horizontal wooden length board was used to measure the length of children 6–23 months of age. On the other hand, a vertical wooden height board was used to measure the height of children aged 24–59 months. Digital seca weight scale was used to measure the weight of the children and read to the nearest 0.1 kg. All measurements were taken twice and the mean was used for analysis. The data collection was supervised and errors were corrected on spot in the field.

### Data analysis

Epi Info software version 3.5.1 was used to enter data and the data were analyzed using SPSS version 20. WHO Anthro software was used to analyze HAZ data and it was compared with NCHS reference standard. Crude association was determined with bivariate analysis and a cut-off point of *p* < 0.25 was used to consider variables for multivariate analysis where confounders were controlled and predictor variables were identified.

## Results

### Socio-demographic characteristics

The response rate of this study was 90%. From the study participant mothers/caregivers, Majority 299 (87.43%) of the mothers/caregivers were married and 246 (71.93%) were protestant in their religion. About two third 224 (65.50%) of mothers and 183 (53.51%) fathers cannot read and write. About 239 (69.88%) of mothers and 244 (71.34%) of fathers were farmers. More than half 188 (54.97%) of the respondents had a family size of above five. Most 294 (85.97%) of the decision on use of money was made jointly (by husband and wife) (Table 1).

**Table 1:** Socio-demographic characteristics of study participants in Sodo Zuria district, South Ethiopia, June 2017

Variables (*n* = 342)		Frequency	Percentage
Maternal Age	15–19	9	2.63
	20–29	192	56.14
	30–39	121	35.38
	≥40	20	5.85
Marital status	Currently married	299	87.43
	Currently unmarried^a^	43	12.57
Ethnicity	Wolaita	336	98.25
	Others^b^	6	1.75
Religion	Protestant	246	71.93
	Orthodox	87	25.44
	Others^c^	9	2.63
Maternal education	Can’t read and write	224	65.50
	Read and write	7	2.05
	Primary education (1–8)	90	26.31
	Secondary education (9–12)	9	2.63
	Above secondary	12	3.51
Paternal education	Can’t read and write	183	53.51
	Read and write	15	4.38
	Primary education (1–8)	113	33.04
	Secondary education (9–12)	11	3.22
	Above secondary	20	5.85
Maternal occupation	Housewife	25	7.31
	Farmer	239	69.88
	Merchant	46	13.45
	Others^d^	32	9.36
Paternal occupation	Farmer	244	71.34
	Government employee	25	7.31
	Merchant	42	12.28
	Other^d^	31	9.06
Monthly Income	<750 ETB	258	75.44
	750–1500 ETB	68	19.88
	>1500 ETB	16	4.68
Decision making on income	Husband	10	2.92
	Spouse	38	11.11
	Jointly	294	85.97
Family size	≤5	154	42.03
	>5	188	54.97
Child sex	Male	164	47.95
	Female	178	52.05
Child age (in months)	6–23	104	30.41
	24–59	238	69.59

^a^widowed, separated, divorced ^b^Amhara, Gurage, Oromo ^c^Muslim, Catholic ^d^non government employee, self employee, student

### Obstetric, maternal and child morbidity related characteristics

Two third 229 (66.96%) of the children were born at term and majority 272 (79.53%) were born in the health facility. Two hundred fifty seven (75.15%) of the children had a normal birth weight. Forty eight (14.03%) mothers were in the age of < 20 years during their first delivery, 286 (83.63%) mothers had ANC follow up, 263 (76.90%) of the mothers did not take any additional meal than usual during their pregnancy and half 172 (51.30%) of the mothers did not use any type of family planning. More than one fourth 89 (26.02%) of the children had diarrheal morbidity in 2 weeks prior to this study, 23 (6.73%) had fever and 20 (5.85%) had cough during the study period (Table 2).

**Table 2:** Obstetric, child morbidity and other maternal characteristics of study participants in Sodo Zuria district, South Ethiopia, June 2017

Variables (*n* = 342)		Frequency	Percentage
Place of delivery	Health facility	272	79.53
	Home	70	20.47
Gestational age at birth	<9 months	14	4.09
	At 9 month	229	66.96
	>9 month	99	28.95
Birth weight	<2500 g	10	2.92
	2500–4000 g	257	75.15
	>4000	75	21.93
Birth order	1	66	19.30
	2–4	160	46.78
	>4	116	33.92
Birth interval from previous	<24 months	69	20.17
	24 months	224	65.50
	>24 months	49	14.33
Still breast feeding	Yes	202	59.07
	No	140	40.93
Reason for not breast feeding	Maternal health problem	19	13.57
	Child refusal	88	62.86
	Pregnancy	33	23.57
Diarrheal morbidity in the last 2 weeks	Yes	89	26.02
	No	253	73.98
Fever	Yes	23	6.73
	No	319	93.27
Cough	Yes	20	5.85
	No	322	94.15
Measles	Yes	8	2.34
	No	334	97.66
Maternal age at first birth	15–19	48	14.03
	20–29	213	62.28
	30–39	70	20.47
	≥40	11	3.22
ANC follow up	Yes	286	83.63
	No	56	16.37
Additional meal during pregnancy/lactation	Yes	79	23.10
	No	263	76.90
Family planning method used	Yes	170	49.70
	No	172	51.30
What type of family planning	Pills	34	20.00
	Depo-Provera	131	77.06
	Others^a^	5	2.94

^a^Norplant, Condom, Intrauterine device

### Child caring practice and environmental health characteristics

Two hundred and twenty eight (66.67%) of the children were initiated on breastfeeding within 1 hour after birth and nearly all 334 (97.66%) of the children were given colostrums. More than half of the children 195 (57.02%) were commenced on complementary feeding at 6 months of age and 264 (77.19%) eat 3 times a day. Source of drinking water for two hundred sixty nine (78.66%) of the households was protected well/spring. Two hundred forty nine households (72.81%) have latrine and 116 (33.92%) dispose waste in open field (Table 3).

**Table 3:** Child caring practice and environmental health characteristics of study participants in Sodo Zuria district, South Ethiopia, June 2017

Variables (*n* = 342)		Frequency	Percentage
Initiation of breast feeding	<1 h after delivery	228	66.67
	1–24 h	105	30.70
	After 24 h	9	2.63
Colostrums feeding	Yes	334	97.66
	No	8	2.34
Pre lacteal food/liquid	Yes	41	11.99
	No	301	88.01
Type of pre lacteal food/drink	Water with sugar	10	24.39
	Butter	4	9.76
	Cow’s milk	27	65.85
Age at initiation of complementary food	1–3 months	32	9.36
	4–5 months	77	22.51
	At 6 months	195	57.02
	After 6 months	38	11.11
Frequency of feeding	<3 times a day	31	9.07
	3 times a day	264	77.19
	>3 times a day	47	13.74
Method of feeding	Hand	156	45.61
	Spoon	124	36.26
	Cup	40	11.70
	Bottle	22	6.43
Variety of food	2 food items	235	68.71
	>2 food items	107	31.29
Leftover food in the household	Given to children	247	72.22
	Given to others	55	16.08
	Throw away	40	11.70
Ever fed child meat	Yes	92	26.90
	No	250	73.10
Completed Vaccination	Yes	327	95.61
	No	15	4.39
Source of drinking water	Protected well/spring	269	78.66
	Unprotected well/spring	35	10.23
	Pipe water	7	2.05
	River	31	9.06
Availability of latrine	Yes	249	72.81
	No	93	27.19
Materials used for hand washing	Water only	21	6.14
	Use soap often	279	81.58
	Use soap always	42	12.28
Waste disposal	Open field	116	33.92
	In pit	138	40.35
	Use as compost	88	25.73

### Prevalence of stunting and its predictors

The prevalence of stunting in this study was 24.9%; 7.9% were severely stunted. The odd of being stunted was about three times higher for females (AOR = 2.8; 95% CI: 1.5, 5.3) and seven times higher for children aged 12–23 months (AOR = 7.1; 95% CI: 2.3, 21.9) compared with children 6–11 months. Children born from mother’s who do not use family planning and children with diarrheal morbidity in the last 2 weeks prior to the study had 2.5 times higher chance of being stunted (AOR = 2.5; 95% CI: 1.1,5.7) and (AOR = 2.5; 95% CI: 1.2,5.3). Children from households with an income of 750–1500 ETB and > 1500 ETB had less chance of being stunted. Children who received pre-lacteal feeding had 3.8 times higher chance of being stunted (AOR = 3.8; 95% CI: 1.2–12.2) (Table 4).

**Table 4:** Factors associated with stunting in Sodo Zuria district, South Ethiopia, June 2017

Variables (*n* = 342)	Stunting	COR	AOR	
	Yes	No	(95% CI)	(95% CI)
Child sex				
Male	30	148	1	1
Female	55	109	1.6 (1.2–2.2)	2.8 (1.5–5.3)*
Child age				
6–11 months	4	28	1	1
12–23 months	20	52	3.8 (2.1–7.2)	7.1 (2.3–21.9)*
24–35 months	22	55	4.2 (2.2–8.1)	6.4 (2.1–19.2)*
36–47 months	21	56	3.8 (2.1–7.0)	3.1 (1.1–9.1)*
48–59 months	18	66	2.6 (1.2–4.7)	2.8 (0.9–7.8)
Educational status of mothers				
Can’t read and write	55	169	1	
Read and write	3	4	0.6 (0.2–1.6)	
Primary education (1–8)	19	71	0.7 (0.5–0.9)	
Secondary education (9–12)	4	5	0.9 (0.4–2.0)	
Above secondary	4	8	0.3 (0.1–0.7)	
Monthly income of the household				
<750 ETB	55	203	1	1
750–1500 ETB	25	43	0.5 (0.3–0.7)	0.2 (0.1–0.5)*
>1500 ETB	3	13	0.2 (0.1–0.4)	0.1 (0.3–0.5)*
Gestational age at birth				
<9 months	10	4	1	
At 9 months	44	185	0.1 (0.3–1.2)	
After 9 months	31	68	0.4 (0.2–0.8)	
Use of family planning				
Yes	41	129	1	1
No	42	130	1.4 (0.8–2.2)	2.5 (1.1–5.7)*
Distance to obtain drinking water				
<15 min’ walk	61	184	1	
15–30 min’ walk	20	64	0.7 (0.5–0.9)	
>30 min’ walk	4	9	0.5 (0.2–1.1)	
Diarrheal morbidity in the last 2 weeks				
Yes	60	193	2.0 (1.4–2.7)	2.5 (1.2–5.3)*
No	25	64	1	1
Family size				
<5	14	140	1	
≥5	71	117	1.8 (1.4–2.4)	
Pre-lacteal feeding				
Yes	75	226	1.2 (0.8–1.9)	3.8 (1.2–12.2)*
No	10	31	1	1

*Significant at *p* < 0.05 for AOR

## Discussion

The prevalence of stunting documented in this study was 24.9%, which is lower than the national prevalence (38%) and the prevalence level of South Ethiopia (39%) [1]. Higher prevalence levels were reported in Eastern Ethiopia (45.8%) [12], Northwest Ethiopia (38.3%) [13], Nigeria (36.7%) [14] and Nepal (38%) [15]. This difference might be because we used NCHS growth standard and those studies used WHO growth standard (which is known to increase the prevalence of under nutrition specially stunting as compared to NCHS growth standard) [16].

Unlike many other study findings, this study documented that the odds of being stunted was higher for females as compared to males. This finding is different from other study findings from Ethiopia, Nigeria, Tanzania and Uganda where males have higher risk of being stunted [14, 17–19]. On the other hand, in favor of our finding a study from Eastern Ethiopia reported that females had higher chance of being stunted [20]. This may be due to cultural issues, gender preference and differential feeding practice which neglects girls [21–23]. Girls are more likely than boys to experience food insecurity due to selective availability of the limited food for boys than girls in Ethiopia [24]. However, a study in India and Pakistan found no statistically significant association between gender and stunting [25, 26].

We have found that the odd of being stunted was higher for children above 12 months as compared to children 6–11 months. Other similar studies conducted in Nigeria, Ethiopia and Pakistan reported that stunting was significantly associated with age above 12 months [26–29]. The trend of stunting peaks for each group of children age up to 35 months and then declines for children 36–47 months of age. The possible explanations are when children are becoming mobile; they are exposed to contaminants in water, food and soil and they ingest them. Inappropriate complementary feeding and introducing children directly to household diet is another possible reason.

Children from middle- and high-income households had less chance of being stunted. This was in agreement with similar study findings in Southwest Ethiopia [30], Northwest Ethiopia [28, 31], Tanzania [18], Nigeria [14] and Nepal [30] where children from the poor wealth quintile had higher odds of being stunted compared with children from the rich wealth quintiles. Children from poorest households had a higher risk of being stunted compared to those from middle- and high-income households [18, 27, 31, 32]. Increased income improves dietary diversity, which in turn improves nutrient intake and nutritional status [33]. Poor wealth status also negatively affects household food access, sanitation and availability of improved water sources.

Children born to mothers who do not use family planning methods had a higher odds of being stunted compared to those mothers who use family planning methods. Mothers who do not use family planning will have large family size and a short birth interval. Studies from South and Southwest Ethiopia [30, 34], Northern Ethiopia [35], Mozambique [36] and Ghana [37] documented that children living in large family size had an increased risk of being stunted as compared to those from small family size. The possible explanations are that having large family size affects intra-household availability of resources and child caring practice. Short birth interval also has an adverse effect on child nutrition and quality of care. It also leads to intrauterine growth retardation, preterm birth and low birth weight which in turn lead to stunting.

In consistence with studies from Nigeria, South and Northwest Ethiopia, children with diarrheal morbidity 2 weeks prior to the study had a higher chance of being stunted [14, 19, 38, 39]. Diarrhea results in poor appetite, digestion problem and malabsorption and malnutrition children on the other hand have severe episodes of diarrhea.

We have found that pre-lacteal feeding was a predictor of stunting. This is consistent with a study from Southern and Northern Ethiopia [19, 39, 40]. The plausible reason may be stunted children are less likely to be introduced to complementary feeding at appropriate age [18]. The other likely reason is that pre-lacteal feeding has a negative effect on breastfeeding which in turn places children at higher risk of under-nourishment.

### Limitations

This study used cross-sectional data. The estimate might be better representative if a longitudinal follow-up data were used. Certain level of recall bias was expected in age estimate of children; the data collectors used different local events to reduce the recall bias.

## Conclusion

The magnitude of stunting in the current study was low. Stunting was significantly associated with being female, age above 12 months, low household income status, mother’s not using family planning and pre-lacteal feeding. Gender-based policies should be enacted in child feeding practice. Interventions should focus on the utilization of family planning and appropriate child caring and feeding practices should be implemented according to the infant and young child feeding recommendations. Water, sanitation and hygiene interventions need to be strengthened to reduce the occurrence of diarrheal morbidity and poverty alleviation interventions should be implemented.

## Abbreviations

ANC: Antenatal care
AOR: Adjusted odds ratio
BMI: Body mass index
CI: confidence interval
COR: Crudes odds ratio
ETB: Ethiopian Birr
HAZ: height-for-age Z-score
NCHS: National child health
SD: Standard deviation
SPSS: statistical package for social sciences
WHO: World health organization
